# The Two Glass Test

**Published:** 1901-10

**Authors:** J. Henry Dowd

**Affiliations:** Buffalo, N. Y.


### The Two Glass Test.

By J. HENRY DOWD, M. D., Buffalo, N. Y.

THE two glass test which is referred to in all works on genito-urinary diseases does not seem to be used to the extent it should, for the reason that its importance as a diagnostic agent is not well explained, neither is the reason given why opacity of the second denotes trouble in the posterior urethra, *i.e.*, behind the compressor muscle. Furthermore, the way in which this test is used by some has been so changed that one is apt to, in fact, cannot help but draw misleading deductions therefrom. That this procedure is very important when dealing with inflammations of the urinary organs cannot be denied, yet certain important points must always be kept in mind, chief among which is the fact that it takes at least two ounces of urine to thoroughly cleanse the anterior urethra of any debris that may have formed therein, and in the case of old inflammatory spots even this will not remove all indications of anterior involvement. With but few exceptions the two glass test can be relied upon to furnish positive information as to involvement of the anterior urethra, posterior urethra, prostate, vesicles, bladder or kidneys. To thoroughly understand why such is possible, the physiology of urination must be understood.

In a perfectly normal condition and eliminating diuretics, changes in the weather, and the like, urine enters the bladder at an average rate of about two ounces every hour. When this viscus becomes filled to a certain extent the retaining power of the internal sphincter gives way and urine enters the posterior urethra, making this a portion of the urinary bladder, the neck. From much careful observation it may be safely stated that this phenomenon occurs on an average of from one and three-quarters to two hours after a previous thorough emptying. Posterior urethritis would not be so commonly diagnosticated as cystitis, providing this was kept in mind, for it must be remembered that excepting physiologically (vesiculitis and congestion of the urethro-vesicle opening in the female accompanied with nervousness) urination sensation in the presence of inflammation is produced by the irritating fluid coming in contact with the inflamed tissue.

The urethra (male) being divided into two parts by the compressor muscle, prevents secretion, which is formed in front from passing backward, and vice versa. It is thus seen that any secretion formed in the posterior urethra cannot pass forward, but as soon as the internal sphincter relaxes, allowing the urine to enter, this (secretion therein) will intermingle and pass backward to become thoroughly mixed with the urine in the bladder. There are exceptions to this rule. Providing the inflammatory action in the posterior urethra is not sufficiently active to produce pus or other debris in sufficient quantities to become detached from the surrounding membrane, it adheres to the same and is washed out by the first gush which also clears the anterior urethra and we find that the second glass may be perfectly clear to the eye. Where the two glass test is expected to give any positive results, two glasses must be used and not one, as is frequently the case, for it is utterly impossible to form any opinion where a patient is allowed to pass a portion of his urine in a glass, and after visual examination, is allowed to pass the second portion in the same receptacle. The progress of inflammation of the urethra, and, for that matter, the whole urinary tract can be more easily studied by the naked eye than by the microscope. In the case of urethritis, where both anterior and posterior urethræ are involved to any extent both glasses will show an opacity in degree according to the amount of trouble present. This can be easily verified by watching the changes taking place in the urine from the onset of an acute specific involvement. As resolution progresses and providing irritation is avoided, it will be noticed that opacity becomes less marked until the first glass will show only debris floating in an almost clear fluid, and the second clear, excepting in certain cases where small comma-like plugs may be seen. Such a condition indicates slight general inflammation of the anterior urethra, being more chronic at certain spots, with very little or no trouble in the posterior urethra, excepting in the presence of plugs which indicate involvement of the prostatic sinuses. Excepting in a very few instances, with the exception of shreds, the same general rule can apply to inflammations of the whole urinary tract.

| Part Involved. | First Glass. | Second Glass. |
|---|---|---|
| Ant. urethritis, acute; p. u. not involved. | Opaque. | Clear. |
| P. u. slightly involved. | Opaque. | Clear, or only slight opacity. |
| P. u. markedly involved. | Opaque. | Opaque, less than first, possibly some blood. |
| P. u. more marked than anterior. | Opaque, possibly shreds. | Same opacity, no shreds. |
| P. urethritis. Ant u. nearly normal. | Opaque, possibly prost. plugs and a few shreds. | Opaque, with more comma-like plugs. |
| Antro-posterior urethritis, prostate involved. | Opaque, some shreds and comma-like plugs. | Opaque, more comma-like plugs than in first. |
| Antro-posterior urethritis with vesiculitis. | Opaque, shreds or no. | Opaque, but more so than first, pus may drop from meatus at end of urination. |
| Antro-posterior urethritis with prostatic abscess. | Opaque, shreds or no. | Same opacity, or may be more marked than first. Free pus may appear at meatus. |
| Stricture with antro-posterior urethritis and involvement of prostatic sinuses. | Slight opacity with comma-like plugs and well-marked shreds of firm consistency. | Almost clear, except comma-like plugs. |
| Acute cystitis, non-tubercular, urethra not involved. | Opaque, no shreds. | Same opacity, often bloody to a marked degree. |
| Chronic cystitis, non-tubercular, urethra not involved. | Opacity resembling hard cider; urine on standing adheres to bottom of glass in compact mass. | Same opacity, occasionally more marked than first. |

| Part Involved. | First Glass. | Second Glass. |
|---|---|---|
| Tubercular cysto-pyelitis. | Opacity of dirty greenish hue; if much blood present, assumes a dirty red; shreds are often seen. | Same. |
| Pyelitis, no urethritis, slight cystitis. | Opaque in degree as to pathological condition present; pus will adhere to bottom of glass on standing; may be of a greenish oily hue. | Same opacity, same phenomena. |
| All inflammation or new tissue formation in urethra absent. | Clear, excepting possibly phosphates, spermatozoa, bacteria, urates, etc., and after passage of sound washings are free of bloody strings. | Same as first. |

378 Franklin Street.

